# Spatial Knowledge via Auditory Information for Blind Individuals: Spatial Cognition Studies and the Use of Audio-VR

**DOI:** 10.3390/s22134794

**Published:** 2022-06-24

**Authors:** Amandine Afonso-Jaco, Brian F. G. Katz

**Affiliations:** 1 Laboratoire Mémoire, Cerveau et Cognition, Université de Paris, 92100 Boulogne Billancourt, France; 2 Laboratoire Développement Individu Processus Handicap Éducation, Université Lumière Lyon 2, 69500 Bron, France; 3 Institut Jean Le Rond d’Alembert UMR 7190, Sorbonne Université, CNRS, 75005 Paris, France

**Keywords:** spatial cognition, spatial audio, blind navigation, binaural audio

## Abstract

Spatial cognition is a daily life ability, developed in order to be able to understand and interact with our environment. Even if all the senses are involved in mental representation of space elaboration, the lack of vision makes it more difficult, especially because of the importance of peripheral information in updating the relative positions of surrounding landmarks when one is moving. Spatial audio technology has long been used for studies of human perception, particularly in the area of auditory source localisation. The ability to reproduce individual sounds at desired positions, or complex spatial audio scenes, without the need to manipulate physical devices has provided researchers with many benefits. We present a review of several studies employing the power of spatial audio virtual reality for research in spatial cognition with blind individuals. These include studies investigating simple spatial configurations, architectural navigation, reaching to sounds, and sound design for improved acceptability. Prospects for future research, including those currently underway, are also discussed.

## 1. Introduction

Spatial cognition is a general term we use to refer to a set of skills that are crucial in our everyday lives, such as representing the space around us and updating that representation as we move, locating, grasping, or pointing to external objects, learning routes, understanding maps, orienting, etc. [1]. It involves two major components: a dynamic one, such as navigation, and a static one, such as memorising the different objects or landmarks localisation, and/or the topographic knowledge [2,3]. In mental representation of space elaboration, there is an important role for peripheral information in updating the relative positions of surrounding landmarks when one is moving [4]. Extracting reliable positional information from multiple objects while moving is likely more difficult without vision as sight allows one to easily locate two or more landmarks in a space at the same time and to build relative references between them by using the triangulation method [5]. In comparison, for a person who is blind, the amount of distal information provided by the environment is reduced and the nature of available landmarks is more difficult to process [6]. Blind people’s perception relies on haptic (tactile-kinesthetic) perception, which requires active exploration of the environment by the individual [7,8]. It involves complex processes that must integrate cutaneous information with proprioceptive and motor information related to the exploration movements [9]. For haptic exploration, mono- or bi-manual, the number of parallel landmarks that can be observed is limited, requiring blind people to collect a sequence of landmarks and to place them all together in their mental map which is, without vision, cognitively more complex, inducing congenitally blind persons to use egocentric coding strategies. Blind people are nevertheless able to acquire spatial information using other non-visual modalities, as shown by their performance in certain spatial tasks, especially when it involves the locomotor system (e.g., [10]). A large number of studies suggest that early visual experience is not a pre-requisite for the acquisition of spatial concepts (for a review, [11,12,13,14]). Blind people are able to mentally generate and correctly manipulate objects and gather spatial information provided by other modalities to create metrically valid internal representations, even though an early experience of vision facilitates the generation and use of mental images [15,16,17,18,19].

What appears to be clear from current literature is that there is no definitive position regarding the influence of the lack of early vision in spatial capacities. When we are interested in spatial cognition, there are as many different aspects that are studied as there are potential variabilities between individuals, due to their time of exposure to visual information, reasons that cause a person to be blind, and influence of both behavioural and cerebral reorganisation related to the visual deficit. Therefore, it is appropriate to focus on some specifics of spatial cognition and to draw conclusions only on those. As an example, in spatial abilities, there is a lot of discrepancy between different studies. Some demonstrate an impairment of early blind person’s capacities: e.g., ref. [20] asked early blind and blindfolded sighted participants to make both direction (pointing) and distance (ratio scaling) judgements. They were invited to judge distances between their home (where the test took place) and other locations (either having been linked via walking by the participants, or otherwise imagined) or between two of these locations, considering themselves being at one of them. Results showed that early blind participants were less accurate in making *direction* estimations, but both groups reached the same level of accuracy in making *distances* judgements. Concerning the results where the location was imagined (lacking the physical memory of being there), the early blind group was less accurate than the sighted one. In a task where participants had to evaluate distances, point to sound sources, and estimate their coordinates, ref. [21] also showed that early blind performed less well. These conclusions are in line with the idea that early visual experience would allow for a more accurate understanding of external spatial coordinate systems [22,23]. In other words, having benefited from an early visual experience contributes to one’s ability in evaluating spatial relationships between distal spatial cues.

However, other results coming from the similar types of studies suggest that primary visual experiences do not influence spatial abilities. Some studies asked participants to estimate the distances between landmarks or objects that had not been linked by a pathway during the initial phase of learning [24,25]. No difference between early blind and blindfolded sighted participants were observed. In the same vein, refs. [26,27] demonstrated that early or congenital blindness has little or no effect on direction and distance estimation of spatial relationships among locations that were actually visited by participants or explored with their fingers on tabletop tasks. A study on sensory substitution in the context of route finding in a simple maze using a sonified/haptified cane providing distance to obstacle information (a mono-aural/non-spatialized distance Geiger counter metaphor [28]), showed comparable performance between blindfolded-sighted and visually impaired individuals [29], supporting the notion that “representation of space is amodal (i.e., modality-independent)”. Repeating the experiment for the same mazes via both physical exploration and a highly simplified desktop navigation showed that while task completion times did not generally improve (spatial memory of the correct route), false turns and associated errors reduced [30].

Focusing on the ability of creating mental representations of space which preserve the topology and metric relations between the different landmarks, suggesting that mental representation correctly informs them about the structure of the space around them, the results regarding blind people are interesting.

In a mental scanning task (originally developed in [31,32]), after a visual, haptic, or visual and haptic learning of a spatial configuration on a tabletop containing five objects, sighted participants performed similarly between the three learning conditions [33]. In the case of the haptic learning condition, all the participant groups (congenitally blind and blindfolded sighted) obtained the result classically obtained in the literature, i.e., a linear relationship between the times of mental exploration and the distances to be mentally explored, and this, without any significant differences between scanning times between groups.

These results are consistent with [11], which employed a mental rotation task with congenitally blind and blindfolded sighted participants, presenting the same conclusions about the non-crucial role of the visual experience for preserving the topology of a spatial configuration.

Other studies, still using a tactile exploration in the learning phase, such as [34], showed the same pattern of results, i.e., the linear relationship between the distance to be mentally travelled and the time needed to do so, for both sighted and blind (from birth or later) participants. Nevertheless, in contrast to [33], blind participants took significantly longer time than blindfolded to perform the task. These results show that even in the case of permanent visual deprivation, blind people were successful in elaborating structured spatial representations, even if this can lead to longer cognitive processing times.

These results are also in line with the work of [35,36] who showed, by employing a task of mental comparison of distances (originally proposed in [32]), that the spatial mental representations elaborated by both the blind (from birth) and the late-blind (temporarily deprived of vision or not) preserved the metric relations, as well as the topological organisation of the different elements composing the initial environment to be learned. However, the time required for the blind participants (both native and late-blind) to solve the task was significantly longer than that required for the sighted. These results support the hypothesis that permanent visual deprivation has an influence on the processes of elaboration of mental representations of space, just as in [37]. These authors have shown in a spatial inference task that participants who were blind from birth and late-blind had similar performances, but that sighted people went significantly faster to solve these tasks. Ref. [38] also interpreted these results as evidence that congenitally blind participants in particular have a slower mental process for solving this type of tasks.

It is interesting to focus on this precise point. One way is to consider that the extra time needed does not reflect a less accurate spatial ability, but more the translation of a more difficult-to-access spatial information.

We can hypothesise that the modality with which an environment is learned influences the metrical properties that the participant infers about the spatial arrangement. In particular, if this modality is more preferred/familiar to blind participants than to sighted ones, such as the haptic modality, the results of [33] are easier to explain compared to those of [34,35,36]. The fact that blind people are slower in the task can be explained by the hypothesis that sighted people directly access a visual representation of the spatial layout and, therefore, only need to “look” at this representation to provide answers. In contrast, people without visual experience must translate the requested information into a more informative (haptic or locomotor) representation by mentally simulating their own movements (walking or “finger running” in a small environment). This “translation” explains why congenitally blind participants required more time to complete the same task.

In the absence of vision, locomotor experience is an alternative source of information for building mental representations of an environment. The onset of independent locomotion has been shown to be a pivotal event in the life of the human infant, triggering surprisingly far-reaching changes in a variety of psychological functions, including coordination of perception and motor skills, spatial cognition, memory, and social and emotional processes. Indeed, moving from one place to another reveals meaningful information about the environment in which one is walking, and all the more so, in the case of blindness [39].

Much work has been performed to investigate the ability of blind people to navigate complex environments without relying on visual information [10,27,40,41]. However, little was known about the nature and structure of the representations blind people use to underpin their navigational performance. Again, image scanning has been shown to be a reliable means of assessing the metric properties of mental images and providing information about the structure of blind people’s mental representations in space.

Ref. [42] investigated several variations of a situation in which sighted participants learned about an environment by walking along paths connecting salient landmarks and then performing a mental scanning task. Results showed that scan time increased with scanned distance, suggesting that the mental representation of space based on locomotor exploration preserves information about the relative distances between different locations. In another experiment, they compared two learning conditions: one in which the path was visually inspected (without locomotion), and the other in which the path was physically walked without seeing it (blindfolded and guided by the experimenter). The scanning task again showed that both learning conditions resulted in the same typical *time* ∝ *distance* correlation, but that absolute scan times were shorter under visual than under locomotor learning conditions. This finding was consistent with the results of studies showing that vision and visual strategies improve the speed and accuracy of spatial performance [1,12,43]. The next step was then to propose the same type of experiment to blind participants in order to clarify whether their mental representations preserved the metric properties of the learned environment. One issue was to distinguish between congenitally blind and late-blind people, since locomotion is by far the most important means by which congenitally blind people learn large spaces.

One way to further investigate these questions is the use of the virtual audio 3D rendering, allowing researchers to construct complex scenes which would be unfeasible in real contexts. The ability to play back individual sounds at specific positions or to create complex spatial audio scenes without having to manipulate physical devices (e.g., silently moving speakers or even walls) offers many advantages. Thus, the use of spatial audio is no more only focused on the study of low-level processes, such as localisation, and is being used as a tool to study higher-level cognitive process.

We would be remiss if we did not mention the sense of *hearing*, and the widespread assumption that blind people have a distinct sense of hearing compared to the sighted population. Numerous studies have been conducted to investigate this claim which focus on a variety of aspects, from spatial precision to reaction time, neural plasticity, and brain activity, (for a review, see e.g., [44]). Although localisation, spectral analysis, and other basic tasks are generally considered of significant importance in understanding basic auditory perception and performance differences between sighted and blind individuals, these performance differences are inherently limited by the capabilities of the human auditory system. Rather, it is how this acoustic and auditory information is used, requiring higher level cognitive processing, where blind individuals can outperform sighted people. An example of where this is evident are navigation tasks. Ref. [45] conducted an experiment where participants walked at a constant distance from a simple straight barrier, being either a wall or a series of 2 m spaced poles, without making physical contact with the barrier. Finger snaps and footfall sounds were the only information. Compared to 14 blind sighted control participants, the 8 blind participants clearly outperformed the control group, some of whom actually considered the task impossible. Results showed that, overall, the blindfolded sighted subjects performance in the *wall* condition was comparable to blind participant performance in the *pole* condition.

With regards to navigation within an architectural space, ref. [46] investigated spatial navigation with sighted and blind children (aged 4.5–9 yrs). In a carpeted room, a tactile landmark was situated in the centre of each of the four walls. In a learning phase, blind or blindfolded participants were guided within the room to the set of landmarks, facilitating the creation of a spatial cognitive map, either with or without the presence of an auditory landmark, i.e., a single metronome ticking at the starting position. Participants then were invited to follow certain trajectories between the tactile landmarks, incorporating familiar paths from the learning phase, as well as novel paths. Results for sighted subjects showed improvements correlated with age, as well as for the auditory landmark condition. Considering only the new paths, all groups benefited from the auditory landmark. In the final *distance* error analysis, the sighted children performed better than the blind subjects in both conditions, with the blind subjects in the condition with the auditory landmark performing comparably to the blindfolded subjects without the auditory landmark. It should be noted that due to the protocol used, it was impossible to separate the effects coming from the auditory landmark or the learning effect.

One can remark the difference in outcomes of the two above cited studies, where, in [45], blind participant children out-performed sighted in auditory obstacle identification, while in [46], blind participants under-performed sighted in a mental map construction through real navigation. Of course, the tasks are rather different in the two, where the first addresses a skill where blind individuals train (unlike sighted individuals) while the second addresses a general skill (mental map construction) via various learning means. As the second study showed improvement for both groups with age, one can assume a potential lag in acquiring this skill by blind individuals, potentially linked to visual-centric notions employed in mental map development at a younger age, ill-suited or more difficult for blind individuals to acquire. This is similar results observed in the study detailed in Section 3 concerning scenario reconstruction after active locomotion.

We present in the remaining sections a review of several experiments conducted using virtual audio 3D rendering to enhance spatial cognition in blind participants, offering direct simulation of a blind individual’s classical interaction with the world. Moving through it and gathering locomotor as haptic feedback and auditory ones. Here, some reports of previous studies conducted in collaboration between researchers from psychology and acoustics on the topic of spatial cognition shedding light on how blind people are offered a better way to investigate their mental spatial representation, their results appears to be quite similar to those of sighted individuals. Section 2 presents a brief introduction to the concepts employed in spatial audio or auditory virtual reality. Section 3 concerns the role of auditory information in assisting the creation of spatial knowledge. Section 4 investigates whether a blind person is able to collect meaningful acoustic information via an audio VR system designed to deliver a realistic 3D experience of the to be learned environment. The review then concludes with perspectives of research, following the same line of inquiry.

## 2. Brief Introduction to Auditory Virtual Reality

Binaural technology is the solution for sound spatialization that is the closest to natural hearing in the real world. Binaural reproduction attempts to mimic the entire set of acoustic cues involved in human localisation of sounds by reproducing the corresponding acoustic pressure at the entrance of the auditory canals via headphones. These two signals are necessarily a complete and sufficient representation of the sound scene, as they represent all the information exploited by the auditory system to identify the 3D position of a sound source. As such, binaural reproduction of spatial information is fundamentally based on the production (via recording or synthesis) of the collection of localisation cues, comprising the ITD (Interaural Time Difference), the ILD (Interaural Level Difference), and monaural spectral cues [47]. Taken together, the effects of these various cues are collected in the so-called Head-Related Transfer Function (HRTF), characterising the spectro-temporal filtering of an incident sound due to the morphology of the listener’s head, torso, and pinnae. The ILD and ITD, varying as a function of source position, are principally determined by the individual’s head size and shape, as well as the position of the ears relative to the head centre.

For more details on this topic, please refer to the following texts [47,48,49,50]. This method is at the foundation of much of today’s virtual reality systems and has been used in the development of navigation guidance systems for the blind (e.g., [51]).

The general motivation for room acoustic modelling has been to enable the construction of acoustically better environments [52]. The most common method is geometrical acoustic, generally employing the technique of *ray tracing*, which models the propagation of sound through an analogy of light rays. In tracing the propagation of thousands, even millions, of rays and their interaction with the surfaces in a complex geometry, the room acoustic response can be estimated. Through such simulations, and subsequent “auralization” of the simulated room acoustic, it is then possible to render *audible* the acoustics of a computer simulated space [53,54]. While employed in the acoustic design of buildings [55,56], the auralization of simulated room acoustics is also at the centre of high quality virtual reality simulations which have been used, in, e.g., multimodal perception research [57,58] and historical reconstructions [59,60,61]. These techniques of binaural audio and room acoustic auralization are employed in several of the studies presented here.

## 3. Comparing Small-Scale Spatial Configuration and Locomotor-Scale Environment on Blind’S Mental Representation Properties

We examine in this set of studies to what extent the size of the environment, as well as the use of an active exploration of this environment by immersion in a virtual world in 3D audio, during the learning phase could have an influence on the metric properties of mental representations. Previous studies examined mental scanning, as well as performance on distance comparison tasks after verbal or haptic learning in blind (congenital or late) and sighted (blindfolded or with unimpaired vision) subjects [15]. The main conclusion was that blind people, like sighted people, were able to generate an accurate mental representation of an environment, which they obtained either by verbal description or by haptic exploration of a small-scale configuration. However, blind people needed significantly more time.

The metric properties of proximal spaces experienced without any motion component might be particularly difficult to encode for congenitally blind people. For them, the optimal conditions for constructing spatial representations are likely to depend on body involvement [27,40,42]. If this is true, it is reasonable to assume that congenitally blind people perform better on tasks in which the acquisition of spatial knowledge is based on locomotion.

The working hypothesis was that mental representations of spatial configurations may be based on different strategies. A sighted person might use mental representations that are iconic in nature, whereas blind persons might better remember sensorimotor contingencies. Ref. [62] have suggested that visual images, even for sighted people, can be representations based on information gathered through a number of different sensory modalities. Ref. [15] elaborated a new experimental context that further opens up the question of how blind people represent space-namely, the role of auditory information in supporting the creation of spatial knowledge [21,63].

**Figure 1:**
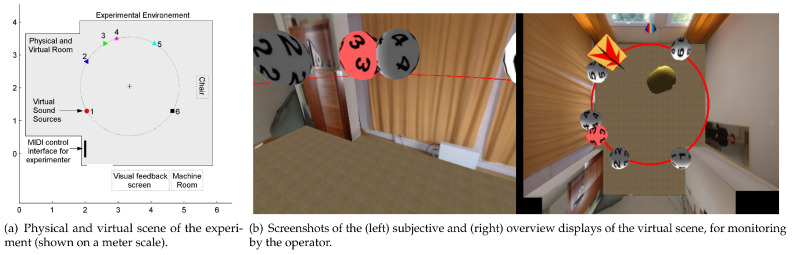
Virtual reality scenario with six sound sources constructed for the mental representation study [64].

The aim was to evaluate the effect of visual experience on mental spatial representation. Two learning conditions were contrasted, one designed to elicit a mental map of an iconic nature, while the other produced a mental one based on perception/action couplings. The comparison was intended to help determine which learning modality produced the most accurate representation of a spatial configuration, as well as to gather information about the perception by blind and sighted people of a world interacting with a three-dimensional audio environment. A large-scale immersive virtual audio environment (absent of visual feedback) with binaural audio and dynamic position and orientation tracking was developed where participants were able to explore and interact with local virtual sound objects (although the boundaries of the physical and virtual worlds aligned, the room acoustics of the space was not represented in the virtual simulation) (see Figure 1). The goal was to investigate mental spatial representations as a function of visual experience. Comparing two learning conditions, the first conceived to elicit a mental map of an iconic nature, the second intended to produced mental maps based on perception/action couplings. This comparison examined which learning modality produced the most accurate spatial configuration representation, in addition to gathering information about the perception by blind and sighted individuals within an interactive three-dimensional audio environment.

**Figure 2:**
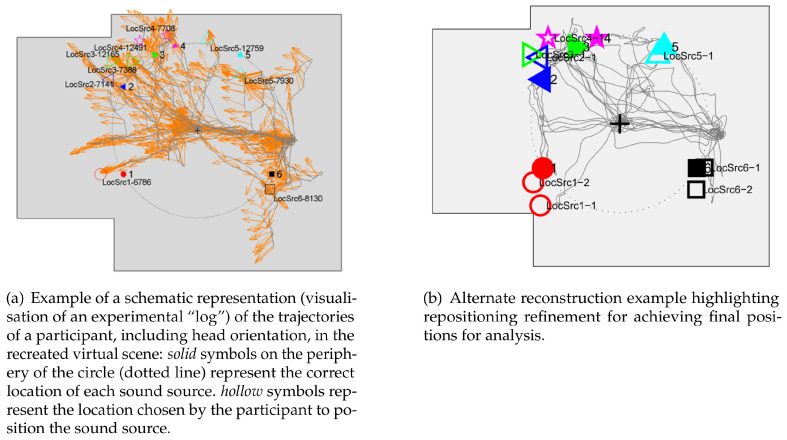
Examples of scenario reconstruction for the mental representation study [44,64].

Participants performed several tasks, including manual reconstruction of the sound scene after a learning phase (see Figure 2) and tasks involving the proposal of different virtual scenes with minor and major metrical changes, followed by questions and the opportunity to correct the scene. Finally, mental scanning and mental distance comparison tasks were proposed [15,36,44].

### 3.1. Protocol

Many previous experiments in the literature employing 3D audio for sound localisation studies or in the development of audio interfaces for blind individuals rely on specialised platforms for spatial rendering of audio with little or no graphical element. Whether using real sound sources [65] or a virtual environment [66], position/orientation user tracking is needed and some concept of a geometrical scene model must be updated in real time. Although the basic components were also present in this study (tracking and scene representation), the selected approach to virtual audio modelling was novel at the time, relying on real-time scene graph incorporating multimedia 3D effects, behaviour modelling, and interaction (VirChor [67]). The rationale was due to the complexity of the experimental setup, requiring the experimenter to monitor the position of both participants and active audio sources. In addition, the protocol’s complexity and its progressive refinement benefited from an open scripting language that could be easily modified by non-programmers. The spatial sound and graphic rendering system architecture and software designs, including elements for dynamically modifying the behaviour of the user interface in response to participant and experimenter inputs are detailed in [64].

The paradigm of scanning and mental comparison of distances was used, with the goal of contrasting verbal and locomotor experiences as sources of learning. This meant that participants had to construct mental maps of more extended full-scale spatial environments, rather than the small configurations previously used in first experiments [35,36]. To this end, we designed an experimental situation suitable for studying blind people’s abilities to construct spatial representations of an environment filled with sounds (see Figure 2). The aim was to evaluate the structural properties of the spatial representations acquired by blind and blindfolded sighted participants in two situations, namely, listening to a verbal description of the locations of different sound sources and physically moving within the environment to spatially locate and position each source.

The procedure consisted of immersing participants, people who are congenitally or late blind and blindfolded sighted participants in a real environment large enough to allow for locomotion. Participants were provided with a virtual audio sound scene, i.e., the virtual experience of a spatial auditory scene was created that consisted of an organised set of natural sources distributed in space. Using the VR platform, the rendered auditory scenes provided participants with the opportunity to interact directly with the different sound sources (approach them, move away from them, walk around them, etc., see Figure 1). The VR platform included a tracking system that captured and recorded participants’ movements as they moved through the environment. The VR tracking system ensured that the virtual auditory scene remained stable in space despite shifts or head movements. Participants reported feeling as if they were surrounded by sound sources perceived at precise positions, creating a coherent spatial environment, as is usually the case in natural environments with real fixed sound sources. Among the advantages of the VR platform was the ability to control the exact geometric position of each sound, which could be changed dynamically without repositioning physical devices. After becoming familiar with the environment using one of two modes (the verbal and locomotor conditions), participants were tested with the scanning and mental comparison of distances paradigm.

### 3.2. Results

The mental scanning results showed that in the case of learning by verbal description, all participants, both blindfolded sighted, late blind or congenitally blind, showed a correlation between mental travel times and distances to be travelled, which was positive and significant. Thus, the greater the distance between two sound sources, the longer the associated travel time, as in the original reference studies [31,68].

In the *verbal* description learning condition, results suggested that in the case of immersion in a full-size 1:1 scale navigable environment, visual experience did not affect mental representations of the configuration, which preserves the metric relations maintained between the different sound sources. Nevertheless, it should be noted that individuals without early visual experience obtained lower correlation coefficients than late-blind and blindfolded sighted individuals.

In contrast, results obtained in the *active exploration* condition showed that for the congenitally blind group, the correlation between distances and mental travel times, although positive, did not reach the significance threshold. In view of the dispersion of the data points, a larger number of participants would seem to be necessary to resolve this question.

Furthermore, results for mental scanning times showed a clear difference in the behaviour for the blind group following the learning of either a small size configuration or a large size environment. Although the classical positive correlation was observed between distances and mental travel times following immersion in the large environment, no time/distance correlation was observed for the small size configuration, for either verbal description or after tactile exploration, reflecting a poor internal organisation of their mental representation.

These results suggest that, in the case of learning the spatial configuration of a locomotor space, early deprivation of visual experience did not have a specific impact. It seems however that early visual experience plays a dominant role in expertise regarding smaller differences, a hypothesis supported by results of the task of mental comparison of distance on small configurations. It was shown that the blind from birth group made significantly more errors in the case of small differences in distances than late blind and blindfolded sighted participants. Furthermore, whereas following learning of a small size configuration, congenitally blind participants took significantly longer than blindfolded sighted participants to compare distances, this was no longer the case after learning the spatial configuration in the immersive environment.

Results obtained with the mental comparison of distance task concluded that regardless of the learning modality of the environment in which they were immersed, all were able to correctly judge the length of two distances, and this independently of the visual experience they may or may not have had. The greater the difference between two distances, the greater the percentage of correct responses, and this for all groups of participants, as seen previously [68]. It was further observed that participants made more errors, and took more time to judge the differences, for small differences in distances, compared to medium or large differences. Response times also showed the same results as those obtained in the literature on smaller configurations, namely that small differences in distance generate longer response times, as compared to medium and large differences in distance [68].

These results suggested that the processes involved in the scanning and mental comparison of distances tasks, although both informing about the preservation of the metric qualities of mental representations, call upon different mechanisms that may or may not be affected, depending on the learning condition. One plausible explanation is that the congenitally blind relied exclusively on other strategies and a form of non-visual spatial imagery to form their mental representations. These strategies would be less effective than the visual imagery used by sighted groups, thus requiring a longer developmental process [69]. In contrast, the larger the initial environment, the less costly this difference in strategy would be, approaching the performance of sighted people in solving these tasks.

From these experiments, one can unequivocally conclude that early visual deprivation does not affect the ability of subjects to correctly represent the environment by preserving their structural isomorphism. In other words, in the case of early visual deprivation, no limits emerged regarding the adaptive capacities to allow the individual to represent environments correctly (i.e., preserving the topology and metric relations between elements). The differences, particularly in response times, suggested that early visual deprivation results in the use of more costly strategies than simply using a mental image, resulting in longer response times. It appears that the *size* of the environment, rather than the learning *modality*, was the determining factor. This alternative explanation deserves more systematic investigation, given the differences between small-scale and large-scale spatial abilities [70].

Regarding the reconstruction of the learned sound scene, the initial hypothesis was that learning through active exploration would provide an advantage for blind participants over learning through verbal description. A second hypothesis involved sighted participants, who were expected to benefit more from verbal description because they were better able to create a visual image of the scene and thus more accurately recreate the original configuration of the scene. The results suggest that active exploration of an environment improves learning of the *absolute positioning* of sound sources compared to verbal description. The same improvement was shown with respect to *radial* distance errors, but only in blindfolded participants. Results demonstrated that both blind participant groups underestimated circle size regardless of learning modality, with mean position error close to zero, and clearly benefited from learning with perception-action coupling.

These results are similar to [20], where blind participants were less accurate in making *direction* estimations, but all groups reached the same level of accuracy in making *distances* judgements. However, they are not in line with others, such as [71] in which blind subjects performed better in estimating the distance to real sound sources by only turning their heads and verbally reporting their position. It is clearly shown that active exploration of the environment improved the performance of blindfolded participants, both in terms of absolute position and size of the reconstructed configuration. It was also observed that subjects who were blind from birth made significantly more errors in angular positioning than late blind or blindfolded groups in both learning conditions. These data are consistent with the results of previous studies dealing with the processing of spatial information in classical real (not virtual) environments [72].

## 4. Wayfinding

Another significant interest in using spatial audio virtual reality is the ability to offer the opportunity to blind people to learn new unknown environments before a real navigation (i.e., going there). Navigation in an enclosed environment requires analysis of a variety of acoustic cues, a task that is well developed in many visually impaired people and for which sighted people rely almost exclusively on visual information. For blind people, creating cognitive maps for spaces such as homes or buildings can be a tedious process.

If one considers the context of a blind individual arriving at a new job site, they could be expect to rely on exploring the building after hours, or at a minimum, when occupancy is very low, to actively explore the architectural environment. The goal of such navigation is to gain some knowledge of spatial configurations and basic characteristics of the acoustic environment, while avoiding being disturbed by other people (including reverberation and echoes, footfall noises, etc.). Later, the individual becomes increasingly familiar with the daily sounds associated with different areas of the environment. The study presented in the following section poses the question of whether a blind individual could gather such acoustic information—critical to subsequent adaptation—using an audio VR system capable of presenting a realistic 3D rendering of the environment of interest. The valuable aspect of a VR system would be the possibility to perform the acquisition phase at one’s own discretion, i.e., in a different place other than the actual environment, e.g., the user’s home or in a public resource centre. In short, is it possible for a person to learn an architectural environment without being physically present? If so, such a system could prove useful for navigational preparation in new and unfamiliar environments.

A comparison of two types of learning was proposed: in situ real displacement versus active navigation in a virtual architecture. In these two conditions, participants were not allowed to use white cane or be accompanied by a guide dog, only acoustic information was available [73].

The study was designed to provide information about the spatial configuration of an enclosed environment through the use of interactive virtual acoustic models. Two interactive 3D acoustic models were created that simulated the two real environments, and an experiment was conducted comparing the mental representations elaborated after real and virtual navigation.

### 4.1. Protocol

Previous observations in the real-world navigation phase have shown that blind individuals make extensive use of self-generated sounds, such as finger snaps and footsteps to determine the position of an object (wall, door, table, etc.).

For that reason, simulation of these self-produced noises was included within the two 3D interactive acoustical models. Regarding the ability of the system to provide an accurate virtual auditory environment, an HRTF selection phase was performed by each individual so that an optimal/individualised binaural rendering could be presented (see [74] for more details on this procedure).

In the experimental condition, participants were given a joystick as a navigation control device and headphones equipped with a head-tracking device. Stepping sounds were automatically reproduced according to the participant’s movement in the virtual environment and corresponded approximately to a 50 step. Navigation speed was continuously variable, ranging from 0.1–1 ms^−1^, proportional to the angle applied to the joystick, i.e., the further it was pushed forward, the faster one advanced, and vice-versa. The finger snap was played as soon as the joystick button was pressed. As movement in the virtual space was limited to a single linear ‘track’, with the ability to only move forward or backwards (all other actions of the joystick were ignored), and head orientation being determined by the actual head orientation of the participant, very little learning time was required for familiarity.

**Figure 3:**
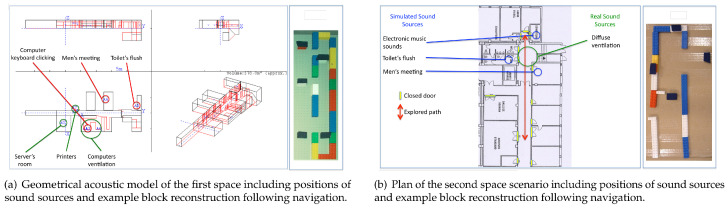
Virtual reality scenarios of the architectural test spaces and example block reconstructions for the architectural perception study [44].

The experimental evaluation consisted of comparing two types of navigation (real and virtual) along the two different corridor environments, with participants given the opportunity to walk back and forth along the path as many times as desired until they were confident enough that they understood the spatial configuration of the environment itself (see Figure 3). In both conditions, all participants stopped several times along the path and listened for elements of the environment. Active, self-generated sounds (their fingers snapping), along with footfall sounds, were reported to be extremely useful for understanding the configuration of specific parts of the environment.

To perform the mental representation assessment tasks, a number of sound sources were placed at specific locations in each environment, and both the sounds and their positions were the same in real and virtual navigation.

Participants were asked to try to mentally represent the configuration of the environment very accurately, taking into account as many of the numerous elements and relative positions as possible (changing floor coverings, openings, windows, doors, objects, obstacles, etc.). In the real condition, two congenitally blind and three late blind subjects (three women, two men) participated in the experiment, while in the virtual condition, four early blind and one late blind individual (three females, two males) explored the same two environments. In order to access the quality of the mental representation blind’s participants elaborated, a mental comparison of distances task between the various sources in the real/virtual environments was employed.

### 4.2. Results

Due to the low number of participants, the results of this study could be described as having low statistical power, nevertheless, results were very informative about the question considered.

The results showed that even at a high level of performance for the real navigation condition, the symbolic distance effect was still confirmed. The probability of making a correct decision when two distances were mentally compared increased with the magnitude of the difference. A similar trend was found in the virtual navigation condition. These results illustrate that the variable real/virtual cannot be considered statistically relevant and that no significant differences were observed between the real and virtual navigation modalities for all three types of distance differences (small, medium, and large). This initial evaluation tended to confirm that both physical displacement (real navigation) and active virtual navigation using a joystick in a virtual architectural acoustic environment enabled blind subjects to create mental representations that preserved the topological and metric properties of the original environment.

Participants also used LEGO^®^ blocks to reconstruct the representation they made from the environment, combined with verbal annotations, according to the mental representation they elaborated from their navigation (real or virtual), see Figure 3. Participants were asked to be as detailed as they could, giving comments concerning the walls, floors, different sound variations, etc.

The results suggest that exploration within an interactive virtual acoustic environment, such as the one developed here, was sufficiently realistic and well defined to provide appropriate information for spatial understanding of the architecture. An interactive virtual simulation could be precise enough so that information about the spatial configuration of the entire environment, not just the positions of sound sources, could be grasped by visually impaired individuals through auditory exploration alone. In short, interactive VR navigation performed well because it included both head tracking and controlled displacement. These results were of particular value because no significant difference was found between the real and virtual navigation conditions in the behavioural measurements. Joystick-controlled navigation enabled participants to build mental representations of indoor spatial structure that included both sound sources and realistic room acoustic simulations. This mental representation preserved the topological organisation and metric properties of the environment, as was the case with real navigation.

## 5. Discussion

The main objective of this paper was to shed light on how spatial audio technology is able to help to investigate spatial cognition, and in particular that of the blind persons, in a new manner. Many previous studies have been constructed to investigate the way primary visual experience can influence spatial cognition, elaborating more or less complex protocols, and leading to controversial results. Particularly in the field of spatial cognition, no one has concluded definitively about the influence of the lack of (early) vision on spatial capacities, likely because of the varied specific aspects that can be studied, including the potential variability between individuals. The major variable being blind congenitally or becoming blind later in life. Numerous studies indicate that early visual experience is not a prerequisite for the acquisition of spatial concepts (for a review, [11,12,13,14]), implying that blind people are able to mentally generate and correctly manipulate objects, gathering spatial information provided by other modalities, to create metrically valid internal representations, even though an early experience of vision facilitates the generation and use of mental images [15,16,17,18,19]. In the presented studies, we focused on a particular spatial ability of blind people, specifically, the ability of creating mental representations of space preserving the topology and metric relations between the different landmarks, suggesting that their mental representation of the configuration correctly informs them about the structure of the space around them, which is crucial in their every day life.

The principle classical tasks informing on this specificity of spatial cognition come from the mental scanning of distance task (originally proposed in [31,75]) and from the mental comparison of distance task (originally proposed by [32], adapted to blind subjects by [36]). After a haptic learning of the configuration, congenitally blind participants’ data displayed the same linear relationship between scanning time and distance as those of (blindfolded) sighted people, the absolute scanning times were not significantly different between sighted and blind people [33], contrary to Kerr [34], who also reported a strong positive correlation between scanning times and the length of mental travel for both participants, but with significantly longer time for blind than for blindfolded persons. This finding suggested that the structure of mental representations of spatial configurations can be achieved despite the absence of sight, although the cost of generating and scanning these representations could be higher for blind people.

Refs. [35,36] showed that the topological organisation and metric relationship between the objects composing a spatial layout were preserved in the mental representations constructed by blind persons in a mental distance comparison task. However, the analysis also showed that the time for mental processing of distances was longer in blind individuals (from birth and later) than in blindfolded individuals, suggesting that definitive visual deprivation affects the way mental representations are processed.

One way to consider the extra time needed by the blind person is to imagine that it is the reflection of a slower mental process of the information, or a more difficult access to spatial information, and not a reflection of a less accurate spatial ability. These differences could be due to the modality under which an environment is learned, and if it is or not a preferred blind modality. This could explain why sometimes blind performances are better or alternatively less accurate than those of blindfolded sighted ones.

In the absence of vision, locomotor experience is an alternative source of information to elaborate mental representations of an environment, but little was known about the nature and structure of the mental representations blind people used to underpin their navigational performance.

One way to investigate this question was the use of the virtual audio 3D rendering, offering direct simulation of an individual’s classical interaction with the world, allowing researchers to construct complex scenes which would be unfeasible in real contexts.

The first study reported here compared small-scale spatial configuration and immersive locomotor-scale environment on mental representation properties, and the role of auditory information in assisting the creation of spatial knowledge. Results of mental scanning times showed a clear difference in the behaviour of blind individuals following the learning of either a small or large scale environment. Although a classical positive correlation was observed between distances and mental travel times following immersion in the large environment, the small size configuration showed no time/distance correlation, either after verbal description or after tactile exploration, reflecting the poor internal organisation of their mental representation. These results suggest that, in the case of learning the spatial configuration of a locomotor space (i.e., one in which one can walk around), early deprivation of visual experience does not have a specific impact, and that early visual experience plays a dominant role in expertise regarding finer differences, supported by results obtained with the task of mental comparison of distances on small configurations. In contrast, once congenitally blind individuals learned the spatial configuration of the immersive environment, they took no more time than blindfolded sighted to compare distances. Thus, our results support the idea that the larger the initial environment, the less costly the difference in strategy between blind and sighted individuals would be, and that it is the scale of the environment, rather than the learning modality, that was critical.

The aim of the second study, using spatial audio virtual reality, was to study the ability to offer the opportunity to blind people to learn via an audio VR system, new unknown environments before a real navigation, offering the opportunity to analyse variety of acoustic cues, offsite, at their discretion, and to elaborate cognitive maps for these spaces before navigating inside.

A comparison was made between two types of learning: real on-site displacement and active navigation in a virtual architecture of two different corridor environments. For both conditions, only acoustic information was available.

Despite the low statistical power of our data due to the low number of participants, results were very informative about the question considered. Using the mental comparison of distance task, we observed the symbolic distance effect and no significant differences between performance in the real and virtual navigation conditions. This initial evaluation tended to confirm that both physical displacement (real navigation) and active virtual navigation with a joystick in a virtual architectural acoustic environment provide appropriate information for spatial understanding and allow blind individuals to create mental representations that preserve the topological and metric properties of the original environment.

These results are valuable as they offer the opportunity to blind persons to learn new environments from their own home before having to go to the real environment. Blindness leads some people to be less social, due to their fear of visiting unknown places. Our results suggest that using audio virtual reality could be of real interest for them. There was, however, a limitation due to the fact that this study was conducted along a corridor path, only allowing participants to go back and forth, linearly, and not allowing them to be free in their exploration.

That is one of the points to be addressed in the context of the RASPUTIN project. One of the research goals is to investigate if a mental map, constructed via a virtual exploration conducted off-site in the privacy of one’s home, could allow blind or visually impaired persons to become well acquainted with a space, such as a new job site, a municipal office, or a museum, prior to actually setting foot in the building, thereby improving the autonomy and sense of security of the individual. Such improvements in mobility could contribute to strengthening self-confidence and increasing access to social and cultural events. To accomplish this, studies are currently being developed to examine the various aspects of the problem, from the perspectives of cognitive science (regarding spatial memory, cognitive mapping, and learning), psycho-acoustics (acoustic cues necessary for spatial comprehension), signal processing (optimisation of room acoustic audio rendering), ergonomics of technology (navigation scenarios and interactivity for improved comprehension), and, finally, with regards to improvements in the individual autonomy of visually impaired navigation (confidence, speed, and precision). Specifically with regards to cognitive maps of architectural spaces, we hope to investigate whether the mental strategies are more egocentric/navigational or allocentric/survey as a function of visual experience [76]. In sighted literature, allocentric perspective is define as global, considering a large environment, frequently termed a “bird’s eye view” of the environment, and is the perspective given by maps. An egocentric perspective refers to the a representation of the environment using the body as reference; the perspective we have when we move through an environment or explore a scene [77,78]. A large body of literature [6,10] suggest that congenitally blind people would be more induced to use egocentric coding strategies when elaborating mental representation of space, and that early visual experience would allow for a more accurate understanding of external spatial coordinate systems [22,23]. The question we want to arise here, is once their mental representation of a new configuration is constructed, without inducing one or another of the strategy (egocentric vs allocentric) to resolve the task in the experimental instructions, will we be able to arrive at the same conclusions.

Finally, the transfer of information from preparatory learning to actual navigation of an architectural site will be investigated, comparing learning the layout of a building via tactile maps or virtual auditory navigation. We will investigate whether either of the cognitive maps developed in the two learning phases improves efficiency or confidence in unaided navigation in unfamiliar locations. These works are related to those of [79,80] or [81] in particular which also seek to allow a more autonomous navigation of blind people in unknown out or indoor environments, but by employing other technologies. Some works are based on the use of cell phones which have the advantage of being within reach of almost everyone [79,80] and, thus, allow the development of interactive virtual guidance applications. This work shows that participants benefit from knowledge acquired via a smartphone-based virtual navigation app on in-situ navigation tasks. However, as soon as they can benefit from a NavCog-type application on the spot, this consequently induces them to rely *more* on the system, rather than on their *a priori* knowledge, such that the use of such virtual navigation aide does not finally allow them to specifically improve their performance. Other authors have shown that navigation in virtual environments, thanks to console controllers [81] in particular, allowed blind people to retrieve information about the environment in a way that would not be possible in the real world (the look-around mode), and that this had an influence on the construction of their mental representations, which were more detailed and contained more information about the space than those of the control group. Thus, regardless of the technologies used, the goal of researchers in this field is to provide a better understanding of the processes involved in the construction of mental representations of space by blind people and how current technologies can enable them to acquire the most relevant information possible to construct reliable mental maps. All these types of works are to be encouraged for a better autonomy and social integration of disabled people.

## Data Availability

Not applicable.
